# Gas Evolution in Operating Lithium-Ion Batteries Studied In Situ by Neutron Imaging

**DOI:** 10.1038/srep15627

**Published:** 2015-10-26

**Authors:** Barbara Michalak, Heino Sommer, David Mannes, Anders Kaestner, Torsten Brezesinski, Jürgen Janek

**Affiliations:** 1 Battery and Electrochemistry Laboratory, Institute of Nanotechnology, Karlsruhe Institute of Technology, Hermann-von-Helmholtz-Platz 1, 76344 Eggenstein-Leopoldshafen, Germany; 2 BASF SE, 67056 Ludwigshafen, Germany; 3 Paul Scherrer Institute, 5232 Villigen, Switzerland; 4 Institute of Physical Chemistry, Justus-Liebig-University Giessen, Heinrich-Buff-Ring 58, 35392 Giessen, Germany

## Abstract

Gas generation as a result of electrolyte decomposition is one of the major issues of high-performance rechargeable batteries. Here, we report the direct observation of gassing in operating lithium-ion batteries using neutron imaging. This technique can be used to obtain qualitative as well as quantitative information by applying a new analysis approach. Special emphasis is placed on high voltage LiNi_0.5_Mn_1.5_O_4_/graphite pouch cells. Continuous gassing due to oxidation and reduction of electrolyte solvents is observed. To separate gas evolution reactions occurring on the anode from those associated with the cathode interface and to gain more insight into the gassing behavior of LiNi_0.5_Mn_1.5_O_4_/graphite cells, neutron experiments were also conducted systematically on other cathode/anode combinations, including LiFePO_4_/graphite, LiNi_0.5_Mn_1.5_O_4_/Li_4_Ti_5_O_12_ and LiFePO_4_/Li_4_Ti_5_O_12_. In addition, the data were supported by gas pressure measurements. The results suggest that metal dissolution in the electrolyte and decomposition products resulting from the high potentials adversely affect the gas generation, particularly in the first charge cycle (i.e., during graphite solid-electrolyte interface layer formation).

Lithium-ion batteries (LIBs) are considered to be the technology of choice for plug-in hybrid and electric vehicles. However, further enhancement in energy and power densities of LIBs is necessary to fulfill the requirements imposed by advanced automotive applications^1^,^2^. One of the main strategies to improve the specific energy is to increase the cathode potential. The vast majority of cathode active materials are oxide intercalation/insertion compounds with different structures, such as spinel-, olivine- and layered-type^3^,^4^. One of them is LiNi_0.5_Mn_1.5_O_4_ (LNMO) spinel, which appears as a promising cathode candidate to be paired with state-of-the-art graphite anodes to achieve high energy density LIBs with good power^5^. The reasons are, among others, the comparably low cost of LNMO as well as its high theoretical specific capacity (147 mAh g^–1^) and high operating voltage (4.7 V vs. Li/Li^+^). There are, nevertheless, several performance limitations that need to be overcome before LNMO/graphite cells become viable for the mass market.

LNMO “half-cells” using Li as anode often exhibit excellent cycling stability, even at elevated temperatures^6^. In contrast, “full-cells” made of LNMO cathode and graphite anode suffer from severe capacity fading upon cycling^7^. In recent years, various degradation mechanisms have been identified and correlated with the performance^8^,^9^; major issues apparently arise from metal dissolution^10^ and gassing as a result of electrolyte decomposition at high potentials^11^,^12^.

Herein, we report on the use of neutron radiography as a non-destructive tool to study gas evolution in operating LIBs *in situ* (exemplified for high voltage LNMO/graphite pouch cells). Although the general principle of neutron and X-ray radiography is quite similar, the results are different. While the X-ray absorption cross-section increases with the atomic number, the neutron absorption cross-section varies non-linearly across the periodic chart of elements^13^. In particular, lithium (^6^Li) and hydrogen strongly scatter neutrons, while, for example, aluminum, carbon and nickel interact only weakly. Because of the high neutron cross-section of the hydrocarbon-based electrolyte solvents employed in LIBs, neutron imaging can be used to visualize decomposition processes, especially those associated with the generation of gaseous products^14^.

Over the years, neutron imaging has become a valuable tool in materials science and electrochemistry^15^. The use of neutron radiography for imaging, e.g., liquid water in gas flow channels of fuel cell membranes^16^,^17^,^18^,^19^,^20^ or ion transport in porous materials^21^ has been demonstrated in many studies. A similar approach can be applied to investigate macroscopic changes occurring inside LIBs. In ^6^Li-containing systems (^6^Li is employed as tracer material), in particular, this method is very effective to study electrode reactions and mass transfer processes, as shown by Kamata *et al.* a few years ago^22^. However, these early investigations were limited by the resolution of the imaging system. In recent years, the through-plane distribution of lithium in graphite-based cells has been presented for different states of charge^23^. Siegel *et al.* showed that neutron imaging using high-resolution detectors is suited for *in situ* quantification of the bulk lithium concentration (on the basis of the optical density)^24^. First dynamic neutron tomography experiments on LIBs have also been performed^25^,^26^,^27^. Furthermore, neutron imaging has been used as a tool to study electrolyte aging/degradation processes^14^,^28^,^29^,^30^. For example, Goers *et al.* investigated the gas evolution (gas area per total electrode area) during solid-electrolyte interface (SEI) formation in LiMn_2_O_4_/graphite cells using a gel-type electrolyte^14^, and Lanz *et al.* showed that excess electrolyte is displaced in the first cycle due to volumetric changes of the active materials^28^.

In this work, we demonstrate that in operando neutron imaging provides both qualitative as well as quantitative information about the gas evolution in LIBs and describe a method to calculate the gas volume. The gassing arising from electrolyte decomposition was followed *in situ* during charging and discharging of pouch cells, with the emphasis placed on the LNMO/graphite system. Cells with different cathode/anode combinations were also studied to highlight the critical role of chemical “cross-talk” between certain types of electrodes. We emphasize that the combination of results from four different cells allows concluding on the individual role of each type of electrode in the gassing process. The “cross-talk” between electrodes in LIBs as such has been reported, e.g., for the case of metal ions migrating from the cathode to the anode, but in principle all products of unwanted side reactions at either anode or cathode will diffuse to the opposite electrode and can trigger additional degradation reactions. In the case of LNMO/graphite, it becomes obvious that the LNMO electrode indeed adversely affects the SEI layer on the graphite anode.

## Results

For the neutron imaging experiments, the pouch cells were mounted in a custom made aluminum holder (see pouch cell setup and holder in Figures S1 and S2 of the Supplementary Information). Neutrons pass through the operating battery and the change in transmission is measured. The result is a two-dimensional radiographic image with dimensions of 152 × 182 mm^2^ (see also Figure S3 of the Supplementary Information).

In the following, we describe the method developed to calculate the gas volume. The beam intensity after attenuation (*I*) by the pouch cell can be approximated using Lambert-Beer’s law according to:


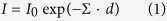


where *I*_0_ is the incident neutron intensity, *Σ* is the material-dependent macroscopic neutron cross-section (or attenuation coefficient), and *d* represents the thickness of the material. To obtain the corrected transmission, the intensity of the beam was normalized as follows:


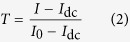


The camera background/dark current (*I*_dc_) and *I*_0_ were determined by measurements with closed shutter and under open beam conditions, respectively.

**Figure 1:**
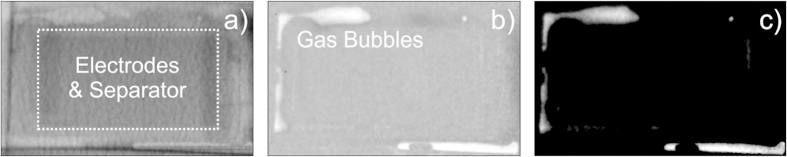
Neutron transmission images of a pouch cell illustrating the different processing steps. (**a**) After noise reduction, (**b**) after normalization to the first image, and (**c**) after segmentation, with grey areas representing the generated gas.

The images were first filtered to remove spots and reduce the noise level (see Fig. 1a). The time series was considered as three-dimensional data volume with two spatial axes and one temporal axis. In so doing, the denoising process produces a more robust estimate of the intensity. The denoising itself was performed using an inverse scale space filter based on the Rudin-Osher-Fatemi model^31^. This particular filter has the characteristic of being edge preserving, while the noise reduction effect is very strong. Overall, the filter model employed here exhibits superior performance compared to convolution filters, median filters and others.

Normalized images were obtained by dividing the corrected transmission (*T*(*t*)) at time *t* by the transmission of the first image (*T*(*t* = 0), pouch cell before cycling). The resulting images (see Fig. 1b) only show local changes occurring upon cycling without the disturbing influence of the topographic contrast. Because of the constant force applied to the electrodes, evolving gases diffuse to regions with lower pressure and therefore the gas bubbles accumulate at the edges of the pouch bag. These images allow for quantitative analysis of the gas evolution. To do so, the neutron transmission data were processed using the software VGStudio MAX. The areas containing gas bubbles were segmented from those without – on the basis of the different pixel grey values – leading to images like that shown in Fig. 1c. These images were then used as a mask to calculate the gas area (on the basis of the noise reduced time series).

**Figure 2:**
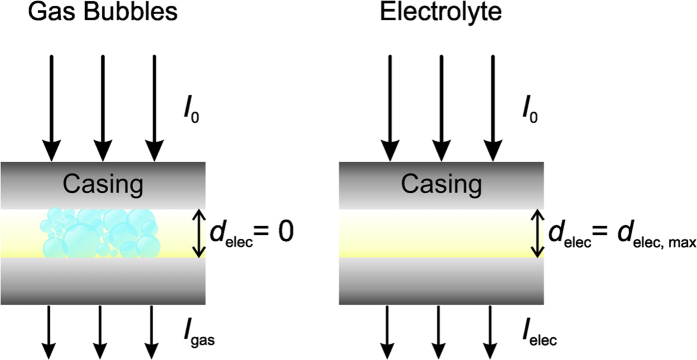
Schematic of the thickness calibration considering areas filled solely with either gas or electrolyte.

For the thickness calibration (see Fig. 2), the thickness of the sample (*d*) is assumed to be constant. The maximum thickness of the electrolyte in the pouch cell was calculated by considering two limiting cases, namely, areas filled with only either gas or electrolyte. The transmission of the brightest (*T*_gas_) and darkest areas of the sample (*T*_elec_) was obtained from the neutron images. The electrolyte thickness at the brightest spot was set to *d*_elec_ = 0 (assumed to have no electrolyte and only gas), while that of the darkest region corresponds to the maximum value (*d*_elec, max_) in the cell.

*d*_elec, max_ was calculated according to:


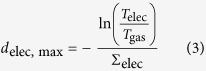


The attenuation coefficient of the electrolyte (*Σ*_elec_ = 5.2385 cm^–1^) was estimated from literature values assuming that the organic electrolyte only consists of ethyl methyl carbonate^32^. The gas bubble thickness at the location *x, y* then corresponds to:


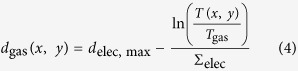


The volume was eventually determined using the known pixel size (*A*_pixel_ = 69 μm × 69 μm) and the mask (*f*) containing the segmented gas bubbles as follows:


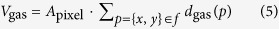


This allows deriving the total amount (volume) of generated gas as a function of time and therefore direct comparison of different battery systems.

In the following sections, we describe the results from neutron imaging. Both first cycle charge/discharge capacities and coulombic efficiency values (see Table S1) as well as movies of the neutron imaging measurements can be found in the Supplementary Information. In addition, gas formation rates are given in Table S2.

**Figure 3:**
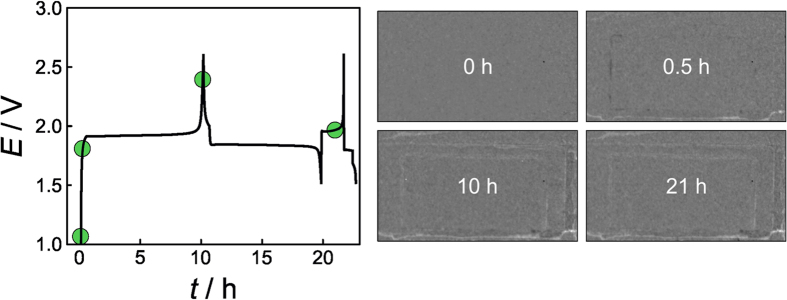
Left: Voltage profiles of an LFP/LTO pouch cell at C/10 (first cycle) and C/2 (second cycle) rates. Prior to first discharge, the battery was kept at open-circuit potential for 30 min. Right: Neutron transmission images taken after different cycling times (indicated by the green dots) during charge and discharge.

Figure 3 shows charge/discharge curves of a LiFePO_4_/Li_4_Ti_5_O_12_ (LFP/LTO) pouch cell together with neutron transmission images taken after different cycling times (see also Supplementary Movie 1). As is evident, negligible gas evolution occurs for this system over the first two cycles. There are, nevertheless, minor changes in transmission, particularly at the electrode edges, which we believe are due to some sort of electrolyte redistribution in the cell. Also, gas trapped inside the pouch bag during cell assembly moves somewhat and appears as bright spots in the images.

Even though the insertion potential range of LTO (1.5 V vs. Li/Li^+^) lies within the electrochemical stability window of most organic electrolytes^33^, gassing of LTO electrodes as a result of electrolyte decomposition at the interface has been reported^34^,^35^. In a recent paper by Gasteiger and co-workers, the gas evolution was attributed to moisture, that is, the presence of trace amounts of water in the cell^36^. No gassing was observed in the first cycle when strictly dried battery components were used, while H_2_ and CO_2_ were detected in cells containing water by on-line electrochemical mass spectrometry. LFP cathodes also operate at a comparably low voltage (3.5 V vs. Li/Li^+^)^37^. Consequently, the LFP/LTO cell system is not supposed to show signs of electrolyte decomposition (note that SEI formation does not occur on LTO)^38^. Here, only the first two cycles are considered and the observation of negligible gas evolution is reasonable given that the pouch cells were assembled inside a dry room.

**Figure 4:**
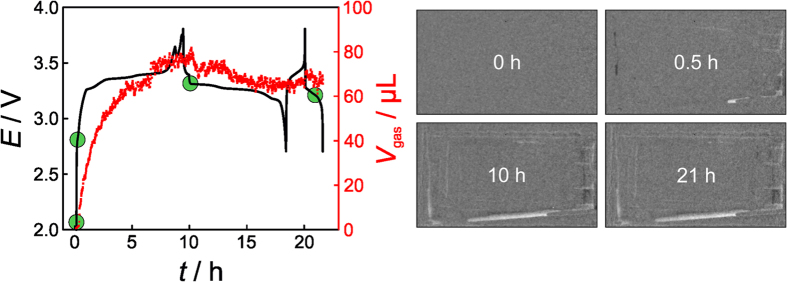
Left: Voltage profiles of an LFP/graphite pouch cell at C/10 (first cycle) and C/2 (second cycle) rates and corresponding gas generation with time. Prior to first discharge, the battery was kept at open-circuit potential for 30 min. Right: Neutron transmission images taken after different cycling times (indicated by the green dots) during charge and discharge.

The voltage profile and amount of generated gas with time as well as representative neutron transmission images of an LFP/graphite cell are shown in Fig. 4 (see also Supplementary Movie 2). The charge plateau at 3.35 V is characteristic of the Fe^2+^/Fe^3+^ redox couple. The other plateaus can be associated with the formation of different lithium-graphite intercalation compounds (note that intercalation occurs in stages)^4^. The spike at the end of the first charge cycle might be indicative of the formation of microdendrites due to lithium plating on the graphite surface. The reason for this is not yet understood.

The neutron images indicate minor gas evolution within the first 0.5 h of charging. However, after 10 h, a significant amount of gaseous products has formed with *V*_gas_ ≈ 70 μL, which remains virtually constant on further cycling. During the first charge cycle, gas generation occurs at high rate due to SEI layer formation arising from electrolyte decomposition on graphite^39^. This surface film is crucial for the battery operation as it protects the anode from deleterious side reactions (e.g., solvent co-intercalation) and prevents further electrolyte decomposition/gassing. Typically, the SEI contains various inorganic and organic/polymeric species^40^. According to Onuki *et al.*, CO and C_2_H_4_ are the major gaseous products, both of which are generated through reduction of ethylene carbonate^11^. However, H_2_ formation due to moisture or, in other words, reduction of water in the electrolyte by the lithiated graphite has also been reported^41^. Taking into consideration that the LFP/LTO cell system showed essentially no gas evolution, we conclude that the gassing solely occurs on the anode and SEI formation on graphite is largely completed after the formation cycle at C/10^41^.

**Figure 5:**
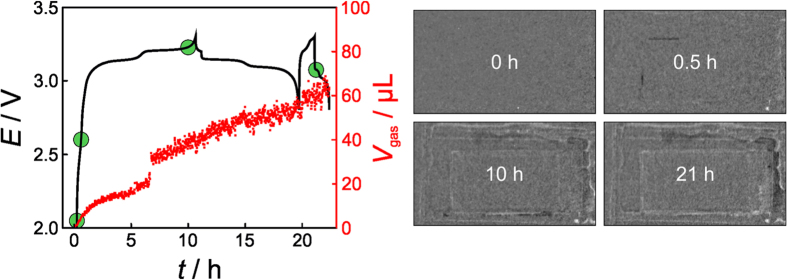
Left: Voltage profiles of an LNMO/LTO pouch cell at C/10 (first cycle) and C/2 (second cycle) rates and corresponding gas generation with time. Prior to first discharge, the battery was kept at open-circuit potential for 30 min. Right: Neutron transmission images taken after different cycling times (indicated by the green dots) during charge and discharge.

The results from neutron imaging of an LNMO/LTO battery pouch cell are shown in Fig. 5 (see also Supplementary Movie 3), indicating that there is gas evolution upon cycling. More specifically, the time resolved data demonstrate little gas generation in the first few hours of charging. However, the rate increases significantly when the second nickel plateau is reached and thereafter remains constant (i.e., *V*_gas_ increases continuously) – the charge profile at C/10 shows two distinct plateaus at 3.13 V and 3.20 V corresponding to the Ni^2+^/Ni^3+^ and Ni^3+^/Ni^4+^ redox couples, respectively. *V*_gas_ is ~65 μL after the first two cycles are completed.

LNMO operates close to the oxidation potential of carbonate-based electrolytes. Thus, electrolyte decomposition is expected when using pristine LNMO. Apparently, a surface film (reminiscent of the graphite SEI) is formed on LNMO, but it is not clear at present whether it is stable and may protect the electrolyte from further decomposition reactions^8^. The gaseous products occurring during surface film formation on LNMO have not been thoroughly investigated yet. However, CO_2_ formation due to oxidation of electrolyte solvents at high potentials has already been reported^42^,^43^. Overall, the neutron imaging data in Fig. 5 demonstrate that the gas generation is continuous and further suggest that the second nickel plateau plays a key role in the gassing behavior of LNMO-based cells.

**Figure 6:**
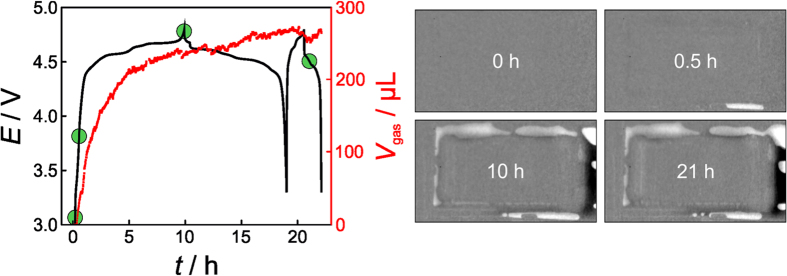
Left: Voltage profiles of an LNMO/graphite pouch cell at C/10 (first cycle) and C/2 (second cycle) rates and corresponding gas generation with time. Prior to first discharge, the battery was kept at open-circuit potential for 30 min. Right: Neutron transmission images taken after different cycling times (indicated by the green dots) during charge and discharge.

Both the cycling and neutron imaging data obtained on the LNMO/graphite system are shown in Fig. 6 (see also Supplementary Movie 4). As is seen, the voltage-time curves show several plateaus and those at 4.57 V and 4.64 V (on charging at C/10) can be attributed to the oxidation of nickel from Ni^2+^to Ni^3+^ and Ni^3+^ to Ni^4+^, respectively^44^. The other plateaus are associated with graphite.

Gas evolution reactions start almost instantly upon charging the pouch cell. The curve shape of *V*_gas_ vs. *t* closely resembles that of LFP/graphite in the beginning and then, after the charge cycle, that of LNMO/LTO. In the first few hours of charging, gaseous products are predominantly generated through reduction of electrolyte solvents. Once this process is completed, gas evolution slows down and further gassing is dominated by oxidation reactions at the LNMO/electrolyte interface.

From the data in Fig. 6, it is apparent that the total amount of generated gas (*V*_gas_ ≈ 250 μL) is larger by a factor of almost 2 than that of LFP/graphite and LNMO/LTO added together. Because a large fraction of the gaseous products is generated during the first charge cycle, this finding suggests that the SEI formation on graphite is somewhat (adversely) affected by the cathode. We believe that both the well-known problem of metal dissolution from LNMO in the electrolyte and formation of detrimental decomposition products at high potentials are responsible for the increased gas evolution. As for the former, the dissolved manganese and nickel ions can be incorporated into the protective surface layer on graphite, thereby poisoning/damaging it. The same may be true for other decomposition products (H^+^, etc.). This leads to continuous SEI formation and gas evolution as well as capacity fading. In recent years, it has been shown that, among others, the HF content in the electrolyte has a profound effect on the metal dissolution^10^. Full-cells with an LTO anode are much less affected by this material specific issue because LTO does not form an SEI. Taken together, the data shown in this section emphasize the need for both high quality “water-free” battery components and additives that allow for the formation of a stable SEI layer on both the cathode and the anode. However, we note that not only the different electrode chemistries but also the potential range during cycling (which of course is linked to the cathode/anode combination) may be responsible for the different gassing behavior, as the electrolyte experiences different electrode potentials.

An interesting question concerns the origin of gas bubbles, i.e., the nucleation sites. One might expect the formation of bubbles directly in the porous anode/cathode architecture and their aggregation between the electrodes – within the separator pores. In fact, we never observed the formation of gas bubbles between the electrodes in cells with good cycling performance, but only in rare cases when the applied pressure was apparently not uniform (see also Figure S4 of the Supplementary Information). Thus, we conclude that bubble formation at the edges is preferred in cells with a homogeneous pressure distribution along the electrodes. This also implies that diffusion of physically dissolved gas in the electrolyte is sufficiently fast.

**Figure 7:**
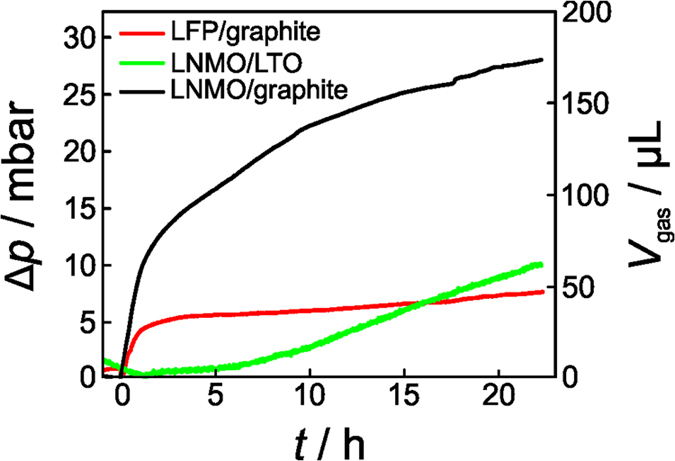
Pressure measurements on LNMO/LTO, LFP/graphite and LNMO/graphite battery cells. Both the pressure increase and calculated gas volume are shown as a function of time. The cycling parameters were identical to those used in the neutron imaging experiments. All values were normalized to an electrode size of 2 × 4 cm^2^.

Pressure measurements on hard-case cells (see photograph and details in Figure S5 of the Supplementary Information) were also performed to corroborate the neutron imaging results. As can be seen from Fig. 7, the pressure curves of all three battery systems showing gas evolution are similar to those determined from the neutron imaging data – they show the very same trend.

The calculation of the amount of generated gas is exemplified for LNMO/graphite in equations (6) and (7). In this case, the measured pressure increase (Δ*p*) was 28 mbar. The dead volume (*V*_dead_) of the hard-case cell was determined to be 6.2 mL. Assuming that the pressure in the pouch cell (*p*_pouch_) is 1 bar, the total amount of gas can be calculated as follows:


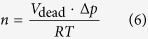



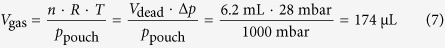


For LNMO/graphite, LNMO/LTO and LFP/graphite, we obtained values of 174 μL, 64 μL and 47 μL, respectively. Compared to neutron imaging, these values are smaller by a factor of ~1.5 (except for LNMO/LTO). Part of the reason for this might be the different cell design used. However, we also note that determining the dead volume by simply weighing the hard-case cell with and without water involves a certain error. Nevertheless, the pressure data confirm the reliability and potential of the method employed here to calculate the gas volume from neutron transmission images.

## Discussion

In operando neutron imaging has been successfully used to quantify the amount of gas generated in lithium-ion batteries as a result of electrolyte decomposition during cycling. Four different pouch cell systems were investigated, namely, LiNi_0.5_Mn_1.5_O_4_/graphite, LiFePO_4_/graphite, LiNi_0.5_Mn_1.5_O_4_/Li_4_Ti_5_O_12_, and LiFePO_4_/Li_4_Ti_5_O_12_. The study of this combination of cells allows concluding on the individual role of cathode and anode in the gassing process. The following conclusions can be drawn:

1. LiFePO_4_/Li_4_Ti_5_O_12_: Gas generation is negligible because the electrolyte has an electrochemical stability window well beyond the potential range of both electrode materials.
2. LiFePO_4_/graphite: Formation of the graphite solid-electrolyte interface layer leads to significant gas evolution during the first charge cycle.
3. LiNi_0.5_Mn_1.5_O_4_/Li_4_Ti_5_O_12_: Continuous gassing due to oxidation of electrolyte solvents on the cathode occurs. The second nickel plateau seems to play a key role, although future work is necessary to confirm this.
4. LiNi_0.5_Mn_1.5_O_4_/graphite: Gaseous products are generated on both electrodes. The total amount of gas is much larger compared to all other battery systems investigated. Poisoning/damaging of the solid-electrolyte interface layer on graphite seems to strongly affect the gassing behavior, particularly in the first charge cycle.

## Methods

The LiNi_0.5_Mn_1.5_O_4_ cathode consisted of 88 wt% active material, 3 wt% conductive carbon (Super C65, Timcal), 3 wt% graphite (SFG6L, Timcal) and 6 wt% polyvinylidene fluoride (PVDF, Kynar HSV 900) and was prepared by the doctor blade method from suspension using a smart coater (Mathis AG, KTF-S). The LiNi_0.5_Mn_1.5_O_4_ loadings were 2.1 mAh cm^–2^ and 1.7 mAh cm^–2^ for full-cells with graphite and Li_4_Ti_5_O_12_ anode, respectively. LiFePO_4_, Li_4_Ti_5_O_12_ and graphite electrodes were purchased and used as received. The LiFePO_4_ loadings were 2.1 mAh cm^–2^ and 1.7 mAh cm^–2^ for full-cells with graphite and Li_4_Ti_5_O_12_ anode, respectively. The Li_4_Ti_5_O_12_ and graphite loadings were 1.6 mAh cm^–2^ and 2.3 mAh cm^–2^, respectively. After drying the electrodes in vacuum at 100 °C overnight, 2 × 4 cm^2^ pouch cells were assembled inside a dry room by stacking anode, polypropylene separator (Celgard 2500) and cathode (single side coated electrodes). The electrolyte used was 300 μL of 1 M LiPF_6_ in a mixed solvent of ethylene carbonate and ethyl methyl carbonate (3:7 by weight, LP57). After electrolyte filling, the pouch cells were vacuum sealed and then mechanically pressed using a custom made aluminum holder.

The battery cells were analyzed in a constant current mode using an Astrol potentiostat (Astrol Electronics AG, Switzerland). After 1 cycle at C/10 was completed, they were charged and discharged at a rate of C/2. The potential ranges were set as follows: LiFePO_4_/graphite, 2.7–3.8 V; LiNi_0.5_Mn_1.5_O_4_/graphite, 3.3–4.8 V; LiNi_0.5_Mn_1.5_O_4_/Li_4_Ti_5_O_12_, 2.8–3.3 V; and LiFePO_4_/Li_4_Ti_5_O_12_, 1.5–2.6 V.

Pressure measurements were performed using the same cell components and cycling parameters, but a hard-case setup with mountable pressure sensor (Omega). The dead volume was determined by weighing the cell with and without water.

Neutron imaging experiments were performed at the Swiss spallation neutron source (SINQ)^45^ using the neutron radiography facility ICON^46^,^47^. This beamline allows for imaging experiments with cold neutrons from a liquid deuterium moderator. The neutron flux at the sample was approx. 1.3 × 10^7^ n cm^–2^ s^–1^ mA^–1^ for a flight tube length of 7.1 m and an aperture size of 2 cm, with wavelengths ranging from 3 to 10 Å^46^. Images were recorded every minute during electrochemical cycling using a CCD camera. The exposure time per image was set at 10 s. The possibility that the gas bubble formation was the result of beam damage was excluded by separate experiments in which only electrolyte and separator were exposed to the neutron beam. The lower detection limit of gas volume was estimated to be ~0.5 μL assuming that the smallest bubble size that can be detected is about 500 pixels in size.

## Additional Information

**How to cite this article**: Michalak, B. *et al.* Gas Evolution in Operating Lithium-Ion Batteries Studied In Situ by Neutron Imaging. *Sci. Rep.* **5**, 15627; doi: 10.1038/srep15627 (2015).

## Supplementary Material

Supplementary Information

Supplementary Movie 1

Supplementary Movie 2

Supplementary Movie 3

Supplementary Movie 4
