# Supplementary material for: Gas Evolution in Operating Lithium-Ion Batteries Studied In Situ by Neutron Imaging
Source: Sci Rep. 2015 Oct 26;5:15627. doi: 10.1038/srep15627 (PMC4620486; doi:10.1038/srep15627)
Supplement: Supplementary Information [file srep15627-s1.doc]

Gas Evolution in Operating Lithium-Ion Batteries Studied In Situ by Neutron Imaging

**Barbara Michalak^1^, Heino Sommer^1,2^, David Mannes^3^, Anders Kaestner^3^, Torsten Brezesinski^1,*^, and Jürgen Janek^1,4,*^**

^1^Battery and Electrochemistry Laboratory, Institute of Nanotechnology, Karlsruhe Institute of Technology, Hermann-von-Helmholtz-Platz 1, 76344 Eggenstein-Leopoldshafen, Germany.

^2^BASF SE, 67056 Ludwigshafen, Germany.

^3^Paul Scherrer Institute, 5232 Villigen, Switzerland.

^4^Institute of Physical Chemistry, Justus-Liebig-University Giessen, Heinrich-Buff-Ring 58, 35392 Giessen, Germany.

*[juergen.janek@kit.edu](mailto:juergen.janek@kit.edu); [torsten.brezesinski@kit.edu](mailto:torsten.brezesinski@kit.edu)


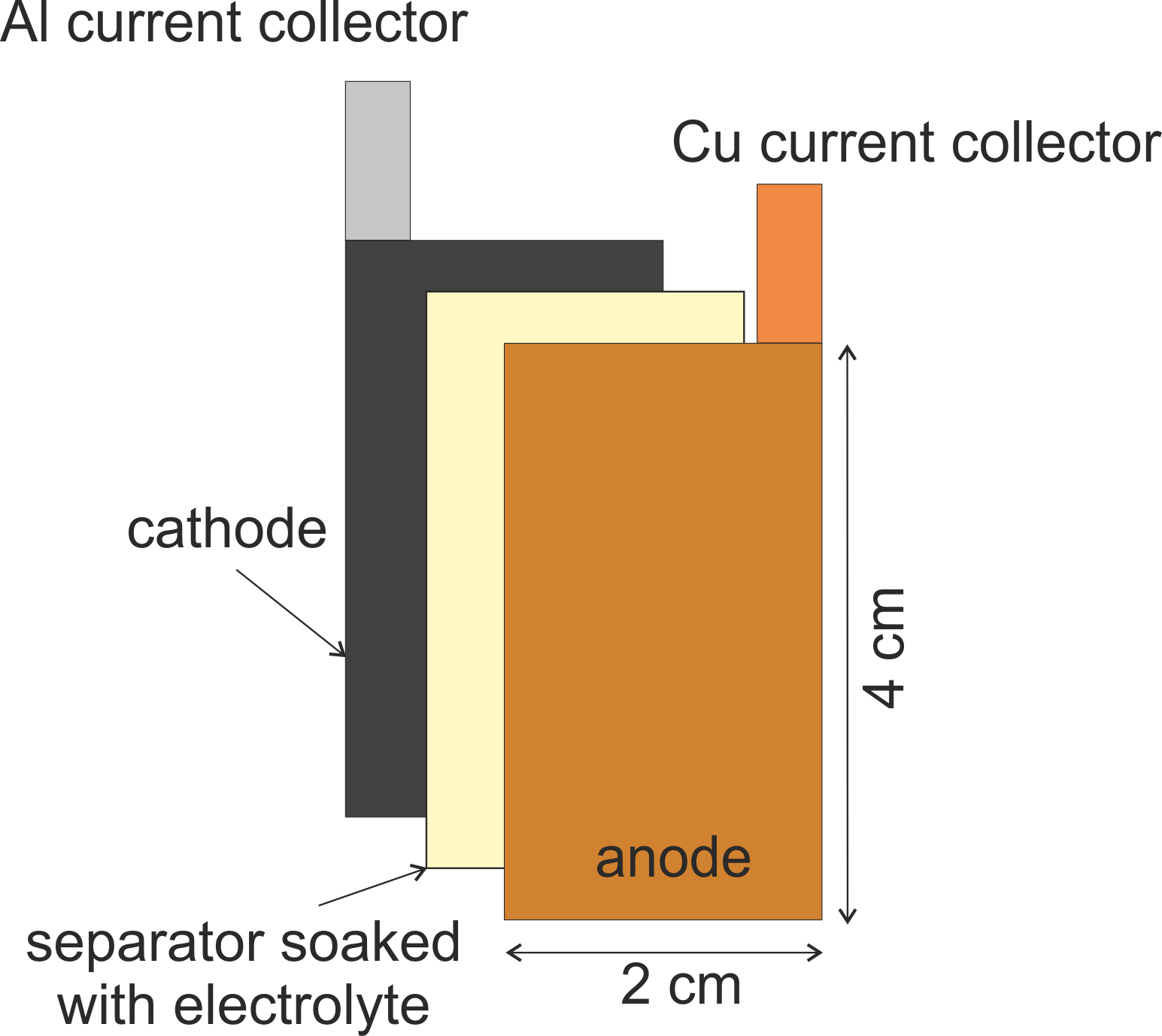


**Figure S1.** Pouch cell setup showing the different battery components.


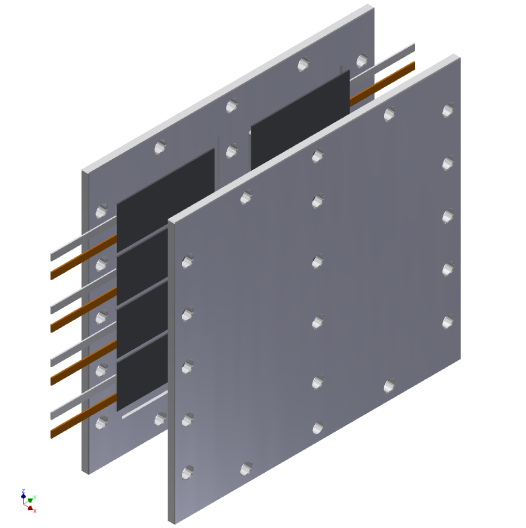


**Figure S2.** Custom made aluminum holder comprising eight pouch cells. The neutron beam passes through the holder/samples and the change in transmission is monitored as a function of time.


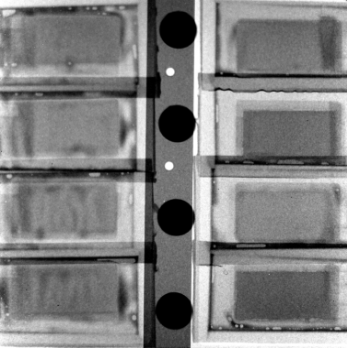


**Figure S3.** As-obtained neutron radiographic image with dimensions of 152 × 182 mm^2^. As can be seen, aluminum interacts only weakly with neutrons.


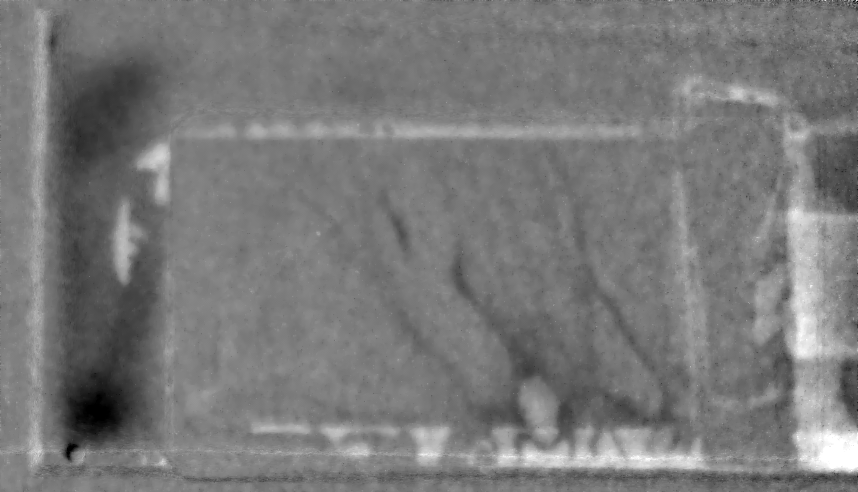


**Figure S4.** Neutron transmission image obtained on an LNMO/graphite pouch cell (after one hour of cycling) showing gas bubbles trapped between the different layers. The presence of gas bubbles between the electrodes is most likely due to non-uniform pressure distribution in the cell and leads to very poor cycling performance.


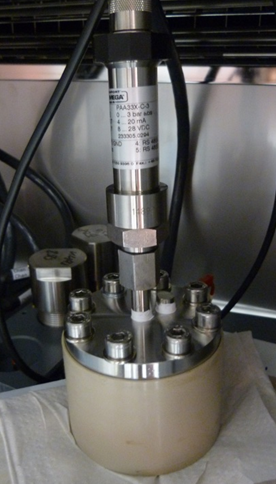


**Figure S5.** Photograph of a typical hard-case cell with pressure sensor. The cells were assembled inside a dry room by stacking anode, glass fiber separator (GF/A, Whatman) and cathode. The electrolyte used was 600 μL of 1 M LiPF_6_ in a mixed solvent of ethylene carbonate and ethyl methyl carbonate (3:7 by weight, LP57).

**Table S1.** First cycle charge/discharge capacities and coulombic efficiency values.

| Cathode/anode combination | Charge capacity / mAh g**^–^**^1^ | Discharge capacity / mAh g**^–^**^1^ | Coulombic efficiency / % |
| --- | --- | --- | --- |
| LFP/LTO | 160.3 | 146.3 | 91.27 |
| LFP/graphite | 157.6 | 142.7 | 90.56 |
| LNMO/LTO | 145.4 | 119.4 | 82.15 |
| LNMO/graphite | 143.5 | 126.5 | 88.11 |

**Table S2.** Gas formation rates derived from the first derivative of the *V*_gas_ curves.

| Cathode/anode combination |  | *t* = 10 min | *t* = 0.5 h | *t* = 5 h | *t* = 10 h | *t* = 21 h |
| --- | --- | --- | --- | --- | --- | --- |
| LFP/graphite | *E* / V | 1.89 | 3.0 | 3.37 | 3.39 | 3.2 |
|  | rate / µL h^−1^ | 0.0396 | 0.0334 | 0.0025 | 0 | 0 |
| LNMO/LTO | *E* / V | 1.35 | 2.50 | 3.14 | 3.23 | 3.29 |
|  | rate / µL h^−1^ | 0.0084 | 0.0074 | 0.0051 | 0.0020 | 0.0034 |
| LNMO/graphite | *E* / V | 2.23 | 3.66 | 4.60 | 4.71 | 4.52 |
|  | rate / µL h^−1^ | 0.0871 | 0.0860 | 0.0148 | 0.0018 | 0.0032 |
